# Patterns of Rotavirus Vaccine Uptake and Use in Privately-Insured US Infants, 2006–2010

**DOI:** 10.1371/journal.pone.0073825

**Published:** 2013-09-16

**Authors:** Catherine A. Panozzo, Sylvia Becker-Dreps, Virginia Pate, Michele Jonsson Funk, Til Stürmer, David J. Weber, M. Alan Brookhart

**Affiliations:** 1 Department of Epidemiology, Gillings School of Global Public Health, University of North Carolina, Chapel Hill, North Carolina, United States of America; 2 Department of Family Medicine, University of North Carolina, Chapel Hill, North Carolina, United States of America; 3 Department of Medicine, Division of Infectious Diseases, University of North Carolina, Chapel Hill, North Carolina, United States of America; The George Washington University Medical Center, United States of America

## Abstract

Rotavirus vaccines are highly effective at preventing gastroenteritis in young children and are now universally recommended for infants in the US. We studied patterns of use of rotavirus vaccines among US infants with commercial insurance. We identified a large cohort of infants in the MarketScan Research Databases, 2006–2010. The analysis was restricted to infants residing in states without state-funded rotavirus vaccination programs. We computed summary statistics and used multivariable regression to assess the association between patient-, provider-, and ecologic-level variables of rotavirus vaccine receipt and series completion. Approximately 69% of 594,117 eligible infants received at least one dose of rotavirus vaccine from 2006–2010. Most infants received the rotavirus vaccines at the recommended ages, but more infants completed the series for monovalent rotavirus vaccine than pentavalent rotavirus vaccine or a mix of the vaccines (87% versus 79% versus 73%, P<0.001). In multivariable analyses, the strongest predictors of rotavirus vaccine series initiation and completion were receipt of the diphtheria, tetanus and acellular pertussis vaccine (Initiation: RR = 7.91, 95% CI = 7.69–8.13; Completion: RR = 1.26, 95% CI = 1.23–1.29), visiting a pediatrician versus family physician (Initiation: RR = 1.51, 95% CI = 1.49–1.52; Completion: RR = 1.13, 95% CI = 1.11–1.14), and living in a large metropolitan versus smaller metropolitan, urban, or rural area. We observed rapid diffusion of the rotavirus vaccine in routine practice; however, approximately one-fifth of infants did not receive at least one dose of vaccine as recently as 2010. Interventions to increase rotavirus vaccine coverage should consider targeting family physicians and encouraging completion of the vaccine series.

## Introduction

Rotavirus gastroenteritis is a leading cause of hospitalizations and emergency department visits among young children in the US [1]. The recently licensed rotavirus vaccines, RotaTeq® (Rotavirus Vaccine, live, oral, pentavalent) [RV5] (Merck & Co., Inc.) and Rotarix® (Rotavirus Vaccine, live, oral, monovalent) [RV1] (GlaxoSmithKline Biologicals), have dramatically reduced incidence of healthcare utilization for rotavirus infection [2]. These vaccines have been recommended for routine use among US infants by the Advisory Committee on Immunization Practices (ACIP) since 2006 [3], [4].

Despite these recommendations, the Centers for Disease Control and Prevention (CDC) estimated that only 67% of eligible children 19–35 months in the US had completed a rotavirus vaccine series in 2011 [5]. Among nine recommended pediatric vaccines assessed by the National Immunization Survey (NIS) in 2011, only the hepatitis A vaccine had lower coverage than the rotavirus vaccine in the US [5], [6]. Little is known about why it can take several years or more for newly recommended vaccines like the rotavirus vaccine to reach high coverage levels, but studies to-date suggest that type of physician visited, geographic residence, socio-economic status, and race may be important predictors [5]–[8]. Considering that the US Department of Health and Human Services (HHS) Healthy People 2020 objectives include vaccinating at least 80% of children with two or more doses of rotavirus vaccine by 2020 and no catch-up schedule for rotavirus vaccines exist, further exploration regarding the determinants of rotavirus vaccine uptake is warranted [9].

Using data from a large population of infants with commercial insurance, we study patterns of use of rotavirus vaccine. We examine individual, provider, and ecologic correlates of rotavirus vaccine use and vaccine series completion. We hypothesize that receipt of other childhood vaccines (e.g., diphtheria, tetanus, and acellular pertussis (DTaP) vaccines) and the type of physician visited will be the most important predictors of rotavirus vaccine series initiation and completion. Our study also examines timeliness of rotavirus vaccine administration as per the 2009 ACIP recommendations and patterns of vaccine uptake from 2006 through 2010 [4].

## Materials and Methods

Infants born in a hospital or outpatient setting between January 1, 2006 and September 30, 2010 were identified from the MarketScan Research Databases (Copyright © Thomson Truven Healthcare, Inc). The MarketScan Research Databases are available for purchase and contain commercial insurance claims data from >111 million individuals in all 50 US states. In 2010, the database included approximately 920,000 infants, corresponding to 25% of the US birth cohort and 50% of the US birth cohort with commercial insurance [10], [11]. Since the data source does not provide birth dates, we used the International Classification of Clinical Diseases, Ninth Revision, Clinical Modification (ICD-9-CM) codes for live born infants, V30–V39, to define the birth date of infants [12]. If an infant had V30–V39 codes on multiple dates, the date of the first code was used as the birth date, and those without such codes and corresponding dates were excluded. Infants with birth dates occurring after administration of rotavirus vaccines, likely due to coding errors, were excluded.

For infants born between January 2006 and February 2010, additional eligibility criteria included having at least eleven months of continuous enrollment in a payer plan captured by our data source. For infants born between March and September 2010, continuous enrollment was defined as enrollment at every month from birth until the end of the 2010 calendar year (the end of available data). In order to ensure adequate follow-up time, only infants born before March 2010 were included in assessments of vaccine series completion.

RV5 and RV1 vaccination status was assessed using the Current Procedural Terminology (CPT) codes, 90680 and 90681. We required infants to have at least one outpatient claim because we thought it was important for our cohort to include only infants that utilized the healthcare system through their private insurance plan to reduce potential misclassification of rotavirus vaccination status. To further reduce exposure misclassification, we excluded infants residing in 13 states with state-funded vaccine programs (Alaska, Idaho, Massachusetts, Maine, North Dakota, New Hampshire, New Mexico, Oregon, Rhode Island, Vermont, Washington, Wisconsin, and Wyoming) except for the cohort of infants used to examine adherence to the recommended vaccine schedule [2].

We used the 2009 ACIP recommendations to assess adherence to the recommended rotavirus vaccine schedule for all calendar years, 2006–2010. If the first dose of rotavirus vaccine was given before the age of six weeks, zero days or after the age of fourteen weeks, six days, then the recommendations were not met. We also considered recommendations to have been violated if any dose was given after the age of eight months, zero days, or if the minimum interval between two doses was less than four weeks.

We calculated simple frequencies, and performed bivariate and multivariable regression analyses using log-risk models that were limited to individual, provider, and ecological characteristics thought to be associated with receipt of at least one dose of rotavirus vaccine, and identifiable in the available data. We also used the same potential individual, provider, and ecological characteristics to explore predictors of rotavirus vaccine series completion. In order to examine whether predictors of rotavirus vaccination changed over time, we repeated the above analyses, restricting the cohort to infants born in 2006 and then 2009. Infants with missing data on any potential predictors were excluded from both of these analyses.

We identified all potential predictors of rotavirus vaccination *a priori*. Individual level variables included sex, DTaP vaccination status (≥1 dose versus 0 doses), number of siblings <10 years old, mother’s age at birth, and overnight hospitalizations prior to the first dose of rotavirus vaccine or by the maximum age at which the first dose of rotavirus vaccine could have been administered as per the ACIP guidelines (14 weeks, 6 days). Variables for race and socioeconomic status were not available. Provider and health plan characteristics included the type of physician visited during ≥70% of the infant’s outpatient visits (pediatrician, family physician, other providers, or no consistent provider type); the network of the care received during ≥70% of the infant’s outpatient visits (in-network, or out-of-network or mixed); and the infant’s type of health plan (basic, comprehensive, high-deductible; Exclusive Provider Organization (EPO) or Preferred Provider Organization (PPO); Health Maintenance Organization (HMO); Point of Service (POS) or POS with capitation; or Consumer Directed Health Plan (CDHP)). All provider and health plan variables were assessed prior to rotavirus vaccination, or fifteen weeks of age if the infant was unvaccinated. Our ecologic factors of interest were region of the infant’s residence (Northeast, Midwest, South, or West) and rurality. In order to better measure rurality, we linked the US Department of Agriculture (USDA), Economic Research Service 2003 rural-urban continuum codes to the claims database via five-digit Federal Information Processing Standard (FIPS) codes. The 2003 rural-urban continuum codes distinguish metropolitan counties by the population size of the metropolitan area, and nonmetropolitan counties by the population size, degree of urbanization, and adjacency to metropolitan areas. These codes classify every US County into either one of three metropolitan categories, or one of six nonmetropolitan categories.

### Ethics statement

This study was considered exempt from human subjects review by the institutional review board at the University of North Carolina.

## Results

### Infant cohorts

Approximately half (51%) of 2.80 million infants identified in the enrollment files between January 2006 and December 2010 had an ICD-9-CM birthing code and corresponding date of service (Figure S1). Infants that were excluded due to missing data generally lacked information on their mother’s age at birth. After additional exclusions, our final cohorts to assess predictors of rotavirus vaccine initiation and completion included 594,117 and 324,264 infants, respectively.

### Temporal trends of rotavirus vaccine uptake

**Figure 1:**
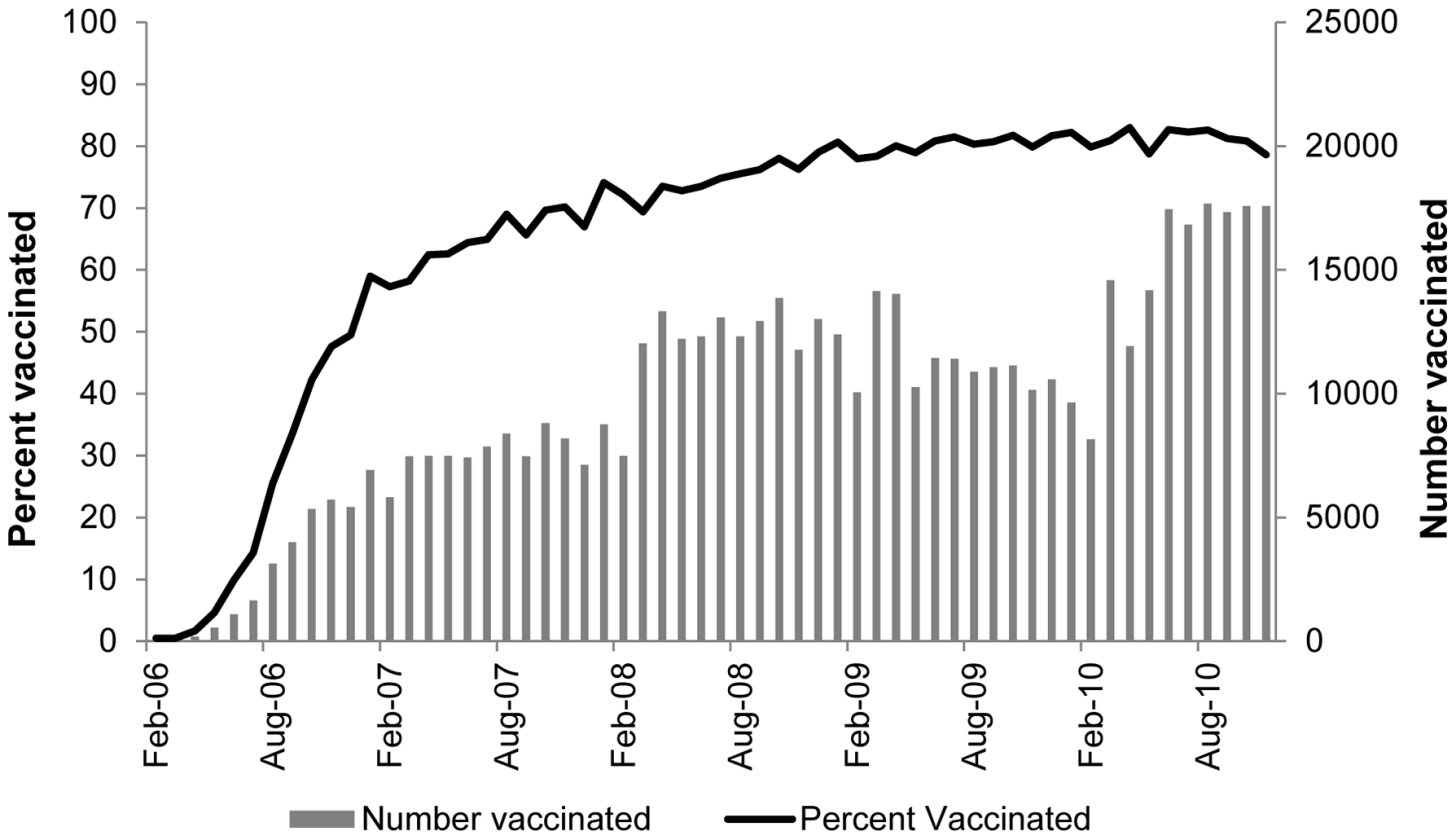
Number and percent of infants vaccinated with ≥1 dose of rotavirus vaccine, February 2006-November 2010 (n = 825,300).

Rotavirus vaccine uptake (≥1 dose) among infants in our cohort increased from 0% when RV5 was licensed (February 2006) to 25% when the first ACIP recommendations were published (August 2006) (Figure 1). Rotavirus vaccine uptake then increased even more rapidly, doubling to 49% by December 2006. The percentage of infants receiving at least one dose of rotavirus vaccine continued to grow steadily, reaching 62% by April 2007 and reaching 70% beginning November 2007. Throughout 2009 and 2010, a median of 81% (range, 78%–83%) of eligible infants were vaccinated with at least one dose of rotavirus vaccine each month. Among the infants receiving a rotavirus vaccine during our study period, 92% received RV5, 5% received RV1, and 3% received a combination of the two vaccines.

### Adherence to the 2009 ACIP recommendations

The median and inter-quartile range of ages at which infants received doses of rotavirus vaccine followed the 2009 ACIP guidelines of two, four, and six months of age (Table 1). Almost all infants received their rotavirus vaccines between the minimum (6 weeks) and maximum (8 months, 0 days) recommended ages, and received dose one and dose two at least four weeks apart. Although the 2009 ACIP guidelines do not specify a maximum interval in which two doses should be given, 18% of infants received a second dose of rotavirus vaccine more than 10 weeks after their first dose, and 7% of infants received their second dose more than 12 weeks after their first dose. Across all years, approximately 8% of infants received their first dose of rotavirus vaccine at ages older than the maximum recommended age for the first dose (14 weeks, 6 days), with 19% of infants in 2006 and 6.0–8.5% of infants from 2007 to 2010, receiving their first dose after age 14 weeks, 6 days. Although most infants who initiated rotavirus vaccination completed the full series, more infants completed the series for RV1 than RV5 or a combination of the two vaccines (87% versus 79% versus 73%, P<0.001).

**Table 1:** Adherence to the rotavirus vaccination 2009 ACIP guidelines (n = 486,295)^1^.

Variable	Number (%)		
Median age in days (IQR)			
Dose 1	63	(61–69)	
Dose 2	126	(123–135)	
Dose 3 (RV5 only)	188	(184–197)	
RV5, number of doses received in series			
One (incomplete)	30,256	(6.8)	
Two (incomplete)	63,294	(14.2)	
Three (complete)	349,599	(78.4)	
Four or more (too many doses)	2589	(0.6)	
RV1, number of doses received in series			
One (incomplete)	3509	(13.5)	
Two (complete)	21,588	(83.3)	
Three or more (too many doses)	823	(3.2)	
Mixed series			
Incomplete	3933	(26.9)	
Complete^2^	9819	(67.1)	
Complete (too many doses)	885	(6.1)	
Administered first dose too early			
(<6 weeks)			
^ ^No	484,979	(99.7)	
^ ^Yes	1316	(0.3)	
Administered first dose too late			
(>14 weeks, 6 days)			
^ ^No	447,442	(92.0)	
Yes	39,557	(8.0)	
Administered any dose too late			
(>8 months, 0 days)			
No	476,647	(98.0)	
Yes	9648	(2.0)	
Minimum interval between first two doses violated (<4 weeks)			
No	450,922	(99.6)	
Yes	1608	(0.4)	

1 Infants vaccinated with RV5, RV1, or a mixed series and enrolled ≥11 months Abbreviations: ACIP, Advisory Committee on Immunization Practices; IQR, interquartile range; RV1, monovalent rotavirus vaccine; RV5, pentavalent rotavirus vaccine.

2 A complete mixed series was defined as receiving 3 rotavirus vaccine doses (≥1 dose of RV1 and ≥1 dose of RV5) when dose 1 and dose 2 were not both RV1.

### Univariate, bivariate and multivariable analyses

Among 594,117 infants, 69% received at least one dose of rotavirus vaccine between February 2006 and December 2010 (Table 2). Most infants in the cohort were also vaccinated with at least one dose of DTaP, were born to mothers 25–39 years of age, were first born children or had one older sibling, visited in-network physicians, were enrolled in EPO or PPO health plans, received outpatient care from pediatricians, resided in the Midwest or South, and lived in large metropolitan areas.

**Table 2:** Estimates of rotavirus vaccine receipt, one or more doses (n = 594,117).

Variable	No. infants receiving ≥1 dose of RV5 or RV1 in category, born 2006–2010 (%)	Bivariate RR, born 2006–2010 (95% CI)	Multivariable RR, born 2006–2010 (95% CI)	Multivariable RR, born 2006 (95% CI)	Multivariable RR, born 2009 (95% CI)
Overall	409,557 (68.9)	–	–	–	–
Sex					
^ ^Female	200,442 (69.0)	Ref	Ref.	Ref.	Ref.
^ ^Male	209,115 (68.9)	1.00 (1.00–1.00)	1.00 (1.00–1.00)	1.00 (0.98–1.02)	1.00 (1.00–1.00)
DTaP vaccination (≥1 dose)					
No	4645 (9.4)	Ref	Ref.	Ref.	Ref.
Yes	404,912 (74.3)	7.91 (7.69–8.13)	7.50 (7.30–7.71)	7.28 (6.59–8.04)	6.95 (6.57–7.34)
Overnight hospitalization					
No	397,832 (69.1)	Ref	Ref.	Ref.	Ref.
Yes	11,725 (64.3)	0.93 (0.92–0.94)	0.96 (0.95–0.97)	1.05 (1.00–1.10)	0.99 (0.98–1.00)
Number of siblings <10 years					
0	187,647 (71.2)	Ref	Ref.	Ref.	Ref.
1	156,922 (68.7)	0.96 (0.96–0.97)	0.97 (0.97–0.97)	0.96 (0.94–0.98)	0.99 (0.98–0.99)
2	52,803 (64.9)	0.91 (0.91–0.92)	0.94 (0.94–0.94)	0.95 (0.92–0.97)	0.97 (0.96–0.97)
3 or more	12,185 (58.3)	0.81 (0.81–0.83)	0.89 (0.88–0.90)	0.91 (0.86–0.96)	0.90 (0.88–0.91)
Mother’s age (years)					
<25	36,376 (63.7)	0.91 (0.90–0.91)	0.95 (0.95–0.96)	0.96 (0.93–0.99)	0.99 (0.98–1.00)
25-<30	130,089 (68.9)	0.98 (0.97–0.98)	0.99 (0.99–0.99)	0.98 (0.96–1.00)	1.00 (1.00–1.00)
30-<35	152,610 (70.4)	Ref	Ref.	Ref.	Ref.
35-40	75,185 (69.2)	0.98 (0.98–0.99)	0.99 (0.98–0.99)	1.02 (0.99–1.05)	0.99 (0.99–1.00)
≥40	15,297 (67.5)	0.96 (0.95–0.97)	0.97 (0.97–0.98)	0.95 (0.90–1.00)	0.98 (0.97–0.99)
Primary provider type					
Pediatrician	266,740 (75.8)	1.64 (1.63–1.66)	1.51 (1.49–1.52)	2.15 (2.02–2.28)	1.35 (1.32–1.37)
Family physician	15,790 (46.1)	Ref.	Ref.	Ref.	Ref.
Other providers	75,312 (61.3)	1.33 (1.31–1.34)	1.31 (1.29–1.32)	1.77 (1.66–1.88)	1.27 (1.25–1.30)
No consistent provider type	51,715 (60.6)	1.31 (1.30–1.33)	1.30 (1.28–1.32)	1.53 (1.43–1.64)	1.23 (1.21–1.26)
Network of provider type					
In-network	368,525 (69.4)	1.07 (1.07–1.08)	1.00 (1.00–1.00)	0.91 (0.88–0.94)	0.99 (0.98–1.00)
Out of network or mix of	41,032 (64.7)	Ref.	Ref.	Ref.	Ref.
networks					
Health plan type					
Basic, comprehensive, or high deductible	7597 (68.0)	0.99 (0.98–1.01)	1.02 (1.01–1.03)	0.76 (0.70–0.83)	1.01 (0.99–1.03)
EPO or PPO	293,141 (68.6)	Ref.	Ref.	Ref.	Ref.
HMO	59,901 (70.5)	1.03 (1.02–1.03)	0.99 (0.98–0.99)	0.90 (0.88–0.93)	1.00 (0.99–1.01)
POS or POS with capitation	36,495 (68.5)	1.00 (0.99–1.01)	0.97 (0.96–0.97)	0.98 (0.95–1.01)	1.00 (0.99–1.01)
CDHP	12,423 (72.9)	1.06 (1.05–1.07)	1.03 (1.02–1.04)	0.95 (0.90–1.01)	1.01 (0.99–1.01)
Region of residence					
Northeast	48,468 (68.2)	0.96 (0.95–0.96)	0.92 (0.92–0.93)	0.73 (0.70–0.76)	0.89 (0.89–0.90)
Midwest	122,396 (66.0)	0.93 (0.92–0.93)	0.98 (0.98–0.98)	0.94 (0.92–0.96)	1.01 (1.01–1.02)
South	202,587 (71.3)	Ref.	Ref.	Ref.	Ref.
West	36,106 (67.9)	0.95 (0.95–0.96)	0.97 (0.97–0.98)	0.72 (0.69–0.76)	0.97 (0.96–0.98)
Type of residence					
Metro with ≥1 M pop	250,066 (71.2)	Ref.	Ref.	Ref.	Ref.
Metro with 250,000 – 1 M pop	74,009 (70.3)	0.99 (0.98–0.99)	1.04 (1.03–1.04)	1.16 (1.13–1.19)	1.02 (1.01–1.02)
Metro with <250,000 pop	39,238 (67.8)	0.95 (0.95–0.96)	1.01 (1.00–1.02)	1.20 (1.16–1.23)	1.00 (0.99–1.01)
Urban with ≥20,000 pop, adjacent to metro area	13,445 (61.9)	0.87 (0.86–0.88)	0.98(0.97–0.99)(	1.02 (0.97–1.07)	0.98 (0.96–0.99)
Urban with ≥20,000 pop,	6348 (56.8)	0.80 (0.78–0.81)	0.93 (0.92–0.94)	0.99 (0.92–1.06)	0.96 (0.94–0.98)
not adjacent to metro area					
Urban with 2500–19,999 pop, adjacent to metro area	15,416 (58.5)	0.82 (0.81–0.83)	0.96 (0.95–0.97)	0.95 (0.91–1.00)	0.96 (0.94–0.97)
Urban with 2500–19,999 pop, not adjacent to metro area	7048 (50.5)	0.71 (0.70–0.72)	0.90 (0.89–0.92)	0.91 (0.84–0.97)	0.94 (0.92–0.96)
Rural or <2500 population, adjacent to metro area	2146 (63.7)	0.89 (0.87–0.92)	0.99 (0.97–1.01)	1.01 (0.90–1.15)	0.99 (0.96–1.02)
Rural or <2500 population,	1841 (56.1)	0.79 (0.76–0.81)	0.98 (0.95–1.00)	1.04 (0.90–1.20)	0.94 (0.90–0.98)
not adjacent to metro area					

Abbreviations: CDHP, Consumer Directed Health Plan; DTaP, diphtheria, tetanus, and acellular pertussis; EPO, Exclusive Provider Organization; HMO, Health Maintenance Organization; Metro, metropolitan; Pop, population; POS, Point of Service; PPO, Preferred Provider Organization; RV1, monovalent rotavirus vaccine; RV5, pentavalent rotavirus vaccine.

The strongest predictors of rotavirus vaccine initiation (≥1 dose) among infants born January 2006-September 2010 were receipt of ≥1 dose of DTaP (multivariable: RR = 7.50, 95% CI = 7.30–7.71), and visiting a pediatrician versus family physician for routine care (multivariable: RR = 1.51, 95% CI = 1.49–1.52). In multivariable analyses, infants were slightly less likely to receive a rotavirus vaccine if they lived in the Northeast as opposed to the South, or in a small urban or rural area as opposed to a large metropolitan area. As the number of siblings less than 10 years of age in the household increased, infants became less likely to receive a rotavirus vaccine.

In order to determine whether predictors of rotavirus vaccine initiation changed over time, we also examined predictors of infants born when RV5 was first licensed (2006) with those born three years after RV5 licensure (2009). In multivariable analyses, compared to the 2006 birth cohort, visiting a pediatrician versus a family physician in the 2009 birth cohort was a less important predictor of rotavirus vaccine initiation (2006: RR = 2.15, 95% CI = 2.02–2.28; 2009: RR = 1.35, 95% CI = 1.32–1.37) as was residing in a metropolitan area with less than one million population versus an area with at least one million population.

**Figure 2:**
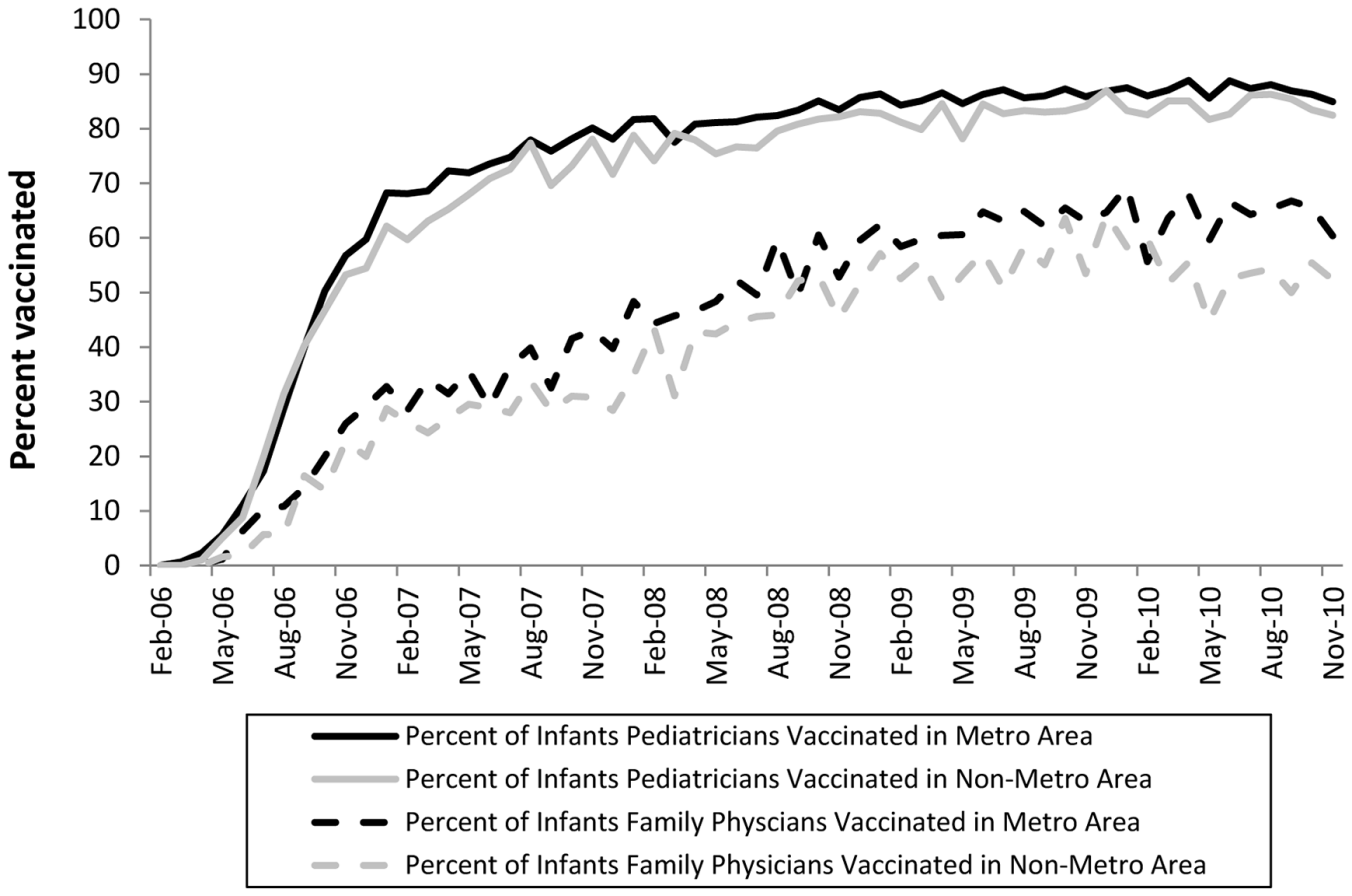
Number and percent of infants vaccinated with ≥1 dose of rotavirus vaccine by physician type and geography^1^ (n = 385,291). ^1^Non-metropolitan geographic areas included any urban or rural designation as defined by the US Department of Agriculture 2003 rural-urban continuum codes, while metropolitan areas included any of the three metropolitan designations.

Family physicians often provide care more frequently in rural areas, and infants visiting family physicians or residing in rural areas were independently less likely to receive a dose of rotavirus vaccine. We therefore explored potential interactions between the type of physician visited (pediatrician versus family physician) for routine care and population size of residence (metropolitan areas versus non-metropolitan areas), but did not find an interaction in these post-hoc analyses (Figure 2).

The most important predictors of rotavirus vaccine series completion were receipt of DTaP and receiving routine care from a pediatrician as opposed to a family physician. The strength of the associations in multivariable analyses were 6-fold and 1.3-fold smaller than in the multivariable analyses of rotavirus vaccine initiation, and the strength of the association decreased from 2006 to 2009 (Table 3). Infants born to younger mothers (<25 years) and with more siblings were slightly less likely to complete the rotavirus vaccine series, and this trend remained consistent in 2006 and 2009. Infants residing outside of metropolitan areas were generally less likely to complete the rotavirus vaccine series. Region of residence was not an important predictor of vaccine series completion.

**Table 3:** Estimates of rotavirus vaccine series completion (n = 324,264).

Variable	No. infants receiving ≥1 dose of RV5 or RV1 in category, born 2006–2010 (%)	Bivariate RR, born 2006–2010 (95% CI)	Multivariable RR, born 2006–2010 (95% CI)	Multivariable RR, born 2006 (95% CI)	Multivariable RR, born 2009 (95% CI)
Overall	259,701 (80.1)	–	–	–	–
Sex					
^ ^Female	127,460 (80.3)	Ref.	Ref.	Ref.	Ref.
^ ^Male	132,241 (79.9)	1.00 (0.99–1.00)	1.00 (0.99–1.00)	0.98 (0.97–1.00)	1.00 (0.99–1.00)
DTaP vaccination (≥1 dose)					
No	2502 (62.6)	Ref.	Ref.	Ref.	Ref.
Yes	257,199 (80.3)	1.28 (1.25–1.31)	1.26 (1.23–1.29)	1.47 (1.32–1.63)	1.24 (1.19–1.29)
Overnight hospitalization					
No	252,144 (80.2)	Ref.	Ref.	Ref.	Ref.
Yes	7557 (77.3)	0.96 (0.95–0.98)	0.97 (0.96–0.99)	1.00 (0.96–1.04)	0.97 (0.95–0.98)
Number of siblings <10 years					
0	120,863 (82.2)	Ref.	Ref.	Ref.	Ref.
1	99,233 (79.5)	0.97 (0.96–0.97)	0.96 (0.96–0.97)	0.96 (0.95–0.98)	0.97 (0.96–0.98)
2	32,500 (76.3)	0.93 (0.92–0.93)	0.92 (0.92–0.93)	0.91 (0.89–0.93)	0.93 (0.92–0.94)
3 or more	7105 (72.4)	0.88 (0.87–0.89)	0.88 (0.87–0.89)	0.88 (0.84–0.92)	0.89 (0.87–0.91)
Mother’s age (years)					
<25	21,999 (73.9)	0.91 (0.90–0.91)	0.91 (0.91–0.92)	0.91 (0.88–0.93)	0.93 (0.91–0.94)
25-<30	81,946 (79.6)	0.98 (0.97–0.98)	0.98 (0.98–0.98)	0.97 (0.95–0.99)	0.98 (0.98–0.99)
30-<35	97,386 (81.5)	Ref.	Ref.	Ref.	Ref.
35-40	48,715 (81.2)	1.00 (0.99–1.00)	1.00 (0.99–1.00)	1.00 (0.98–1.02)	1.00 (0.99–1.01)
≥40	9655 (80.1)	0.98 (0.97–0.99)	0.98 (0.97–0.99)	0.97 (0.93–1.01)	0.98 (0.97–1.00)
Primary provider type					
Pediatrician	171,512 (82.0)	1.16 (1.14–1.17)	1.13 (1.11–1.14)	1.23 (1.16–1.31)	1.14 (1.12–1.16)
Family physician	8554 (70.9)	Ref.	Ref.	Ref.	Ref.
Other providers	48,874 (77.4)	1.09 (1.08–1.11)	1.07 (1.06–1.08)	1.17 (1.10–1.24)	1.09 (1.07–1.11)
No consistent provider type	30,761 (77.1)	1.09 (1.07–1.10)	1.07 (1.06–1.08)	1.18 (1.11–1.25)	1.08 (1.06–1.10)
Network of provider type					
In-network	234,753 (80.1)	1.01 (1.00–1.01)	1.00 (0.99–1.00)	1.00 (0.97–1.03)	0.97 (0.96–0.99)
Out of network or mix of	24,948 (79.9)	Ref.	Ref.	Ref.	Ref.
networks					
Health plan type					
Basic, comprehensive, or high deductible	3639 (81.0)	1.01 (1.00–1.03)	1.02 (1.00–1.03)	0.98 (0.91–1.04)	1.01 (0.99–1.03)
EPO or PPO	183,987 (79.8)	Ref.	Ref.	Ref.	Ref.
HMO	40,726 (80.9)	1.01 (1.01–1.02)	1.00 (1.00–1.01)	1.02 (1.00–1.04)	0.99 (0.98–1.00)
POS or POS with capitation	24,960 (80.2)	1.01 (1.00–1.01)	1.00 (1.00–1.01)	1.02 (1.00–1.05)	0.99 (0.98–1.01)
CDHP	6389 (81.2)	1.02 (1.01–1.03)	1.01 (1.00–1.02)	1.04 (0.99–1.08)	1.00 (0.99–1.02)
Region of residence					
Northeast	29,415 (80.7)	1.01 (1.01–1.02)	0.99 (0.99–1.00)	1.05 (1.02–1.08)	0.97 (0.96–0.98)
Midwest	78,228 (80.8)	1.01 (1.01–1.02)	1.02 (1.02–1.03)	1.01 (1.00–1.03)	1.03 (1.02–1.03)
South	131,635 (79.8)	Ref.	Ref.	Ref.	Ref.
West	20,423 (78.5)	0.98 (0.98–0.99)	0.99 (0.98–0.99)	0.93 (0.90–0.97)	0.99 (0.98–1.00)
Type of residence					
Metro with ≥1 M pop	160.617 (81.3)	Ref.	Ref.	Ref.	Ref.
Metro with 250,000 – 1 M pop	47,204 (81.1)	1.00 (0.99–1.00)	1.01 (1.00–1.01)	1.01 (0.99–1.03)	1.01 (1.00–1.02)
Metro with <250,000 pop	24,533 (77.7)	0.96 (0.95–0.96)	0.98 (0.97–0.98)	0.96 (0.94–0.99)	0.98 (0.97–0.99)
Urban with ≥20,000 pop, adjacent to metro area	8095 (76.1)	0.94 (0.93–0.95)	0.96 (0.95–0.97)	0.87 (0.83–0.92)	0.99 (0.97–1.01)
Urban with ≥20,000 pop,	3869 (74.7)	0.92 (0.90–0.93)	0.94 (0.94–0.96)	0.91 (0.86–0.97)	0.95 (0.92–0.97)
not adjacent to metro area					
Urban with 2500–19,999 pop, adjacent to metro area	9000 (73.5)	0.90 (0.89–0.91)	0.93 (0.92–0.94)	0.91 (0.87–0.95)	0.94 (0.92–0.95)
Urban with 2500–19,999 pop,	3997 (70.0)	0.86 (0.85–0.88)	0.90 (0.88–0.91)	0.84 (0.78–0.90)	0.90 (0.87–0.92)
not adjacent to metro area					
Rural or <2500 population, adjacent to metro area	1333 (77.3)	0.95 (0.93–0.97)	0.98 (0.95–1.0)	0.94 (0.84–1.04)	0.98 (0.94–1.03)
Rural or <2500 population,	1053 (71.7)	0.88 (0.85–0.91)	0.92 (0.89–0.95)	1.03 (0.92–1.15)	0.92 (0.87–0.98)
not adjacent to metro area					

Abbreviations: CDHP, Consumer Directed Health Plan; DTaP, diphtheria, tetanus, and acellular pertussis; EPO, Exclusive Provider Organization; HMO, Health Maintenance Organization; Metro, metropolitan; Pop, population; POS, Point of Service; PPO, Preferred Provider Organization; RV1, monovalent rotavirus vaccine; RV5, pentavalent rotavirus vaccine.

## Discussion

We observed rapid diffusion of the rotavirus vaccine into routine practice shortly after licensure in the US. Approximately three quarters of infants born from early 2008 through mid-2010, received two or more doses. This estimate is slightly higher than the CDC estimate that analyzed data for infants born during approximately the same time period using a population-based telephone survey (NIS), and 5% lower than the HHS’ Healthy People 2020 goal [5], [9]. Our estimate may be higher than the CDC estimate and may have overestimated the progress towards the Healthy People 2020 goal for several reasons. First, our population included only infants with commercial insurance who may be more likely to be vaccinated than other infant populations, such as the uninsured or those with Medicaid insurance. Second, our cohort consisted of a non-population based sample of infants. Since the MarketScan Research Databases have increased in size over time, our data were weighted towards the later years (e.g., 2010) when rotavirus vaccine coverage was relatively high compared to the earlier years. In addition, infants residing in rural and small urban areas were less likely to be vaccinated in our study, but also underrepresented.

It was surprising that one-quarter of eligible infants received at least one dose of rotavirus vaccine prior to the publication of the first ACIP recommendations in August 2006. This reflects the importance of other communication networks and the apparent readiness of the manufacturer, insurance companies, and providers to deliver the rotavirus vaccine. Despite the initial rapid uptake of the rotavirus vaccine, approximately one-fifth of infants were still not receiving the vaccine in January 2009 and coverage has failed to further increase since this time. Education interventions, particularly those targeted at family physicians should be considered. This recommendation is consistent with the results of a 2007 nationally-representative survey of pediatricians and family physicians which found that pediatricians were much more likely to administer the rotavirus vaccine to eligible infants than family physicians, possibly because family physicians were more concerned with vaccine safety and adding additional vaccines to the childhood schedule than pediatricians [8]. Studies examining other vaccines in various populations of infants and young children have also shown that family physicians may be less likely to adopt and may be less knowledgeable about vaccine recommendations than pediatricians [13].

Since most children who received a rotavirus vaccine also received at least one other recommended childhood vaccine (e.g., DTaP), it appears that neither parents nor providers are “cherry-picking” vaccines. Rather, it appears that infants either generally receive the recommended childhood vaccines or do not. This observation is further supported by a post-hoc analysis that found a high correlation between the number of doses of DTaP (one, two, or three) and number of doses of RV5 (one, two, or three) received among infants in our cohort (r = 0.76). Since our cohort consisted of infants with private insurance who had at least one outpatient record, failure to access the healthcare system cannot fully explain why some infants did not receive recommended vaccines, such as DTaP or rotavirus. Based on our results, interventions aimed at increasing the coverage of any one childhood vaccine may help increase the coverage and timeliness of other recommended childhood vaccines, assuming that vaccine availability is not an issue. This was shown to be the case for the DTaP vaccine in Australia, where the third dose coverage of DTaP vaccine in a pre-RV5 cohort was 80%, but increased by 5 to 12 percent once the RV5 vaccine was available and widely used [14].

Overall, adherence to the 2009 ACIP guidelines for rotavirus vaccine administration was high. Although we compared all years of data (2006 to 2010) to the 2009 ACIP guidelines which are less stringent than the 2006 ACIP guidelines, adherence remained high even when we reanalyzed the 2006–2008 data using the 2006 ACIP guidelines (data not shown). Despite overall high levels of compliance to the 2009 ACIP recommendations, ensuring that infants complete the rotavirus vaccine series could be improved. Other multi-dose vaccines face a similar challenge. Prior to rotavirus vaccine availability, the vaccination histories of over 17,000 children in the 2005 NIS were reviewed, revealing that of the 28% of children not compliant with ACIP recommendations, two-thirds were categorized as such because they were missing doses for multi-dose vaccinations [15]. However, since vaccination coverage has been shown to increase as the number of physician office visits increase, one remedy physicians could consider is vaccinating infants at-risk for missing office visits with RV1 since it requires only two doses to complete the series [16]. However, since identifying infants at-risk for missing office visits can be difficult, this recommendation may only be practical in theory. Furthermore, post-marketing data comparing partial series effectiveness of RV5 to RV1 are limited [17].

In addition to the limitations already discussed, our analyses are subject to the following additional limitations. First, many variables potentially predictive of rotavirus vaccine uptake were not available in our data. Further research is needed to examine the effect of potentially relevant predictors, such as race, ethnicity, family economic status, and physician reimbursement levels. Second, we were unable to validate important estimated dates, such as birth dates and rotavirus vaccination dates. While such misclassifications could affect the results of our analysis that assesses adherence to the 2009 ACIP recommendations, we do not suspect that there was enough misclassification to affect our overall conclusions and they are consistent with the results from another recently published study [18]. Third, while we do not suspect that the factors predicting RV5 uptake would differ from the factors predicting RV1 uptake, our results mainly reflect patterns of RV5 use since 92% of the infants in our cohort exclusively received this vaccine. Finally, the infants in our cohorts were not representative of the US infant population; however, our study included nearly 600,000 infants with commercial insurance who may represent the group of infants that most commonly utilizes the rotavirus vaccines.

## Conclusion

Our study revealed rapid initial uptake of the rotavirus vaccine after licensure of RV5. However, even several years after licensure, many children still did not receive the vaccine or received an incomplete series. Quality improvement efforts should focus on ensuring that (1) infants complete a rotavirus vaccine series; (2) family physicians receive the adequate education and support necessary to increase the rates of vaccination among infants in their care; and (3) other recommended infant vaccinations are administered.

## Supporting Information

Figure S1 Development of study cohorts, MarketScan Research. Databases, 2006–2010 Abbreviations: Dec, December; ICD-9, International Classification of Diseases, Ninth Revision; Jan, January; RV, rotavirus; Sep, September.
(TIF)
