# Supplementary figures and images for: Patterns of Rotavirus Vaccine Uptake and Use in Privately-Insured US Infants, 2006–2010
Source: PLoS One. 2013 Sep 16;8(9):e73825. doi: 10.1371/journal.pone.0073825 (PMC3774785; doi:10.1371/journal.pone.0073825)

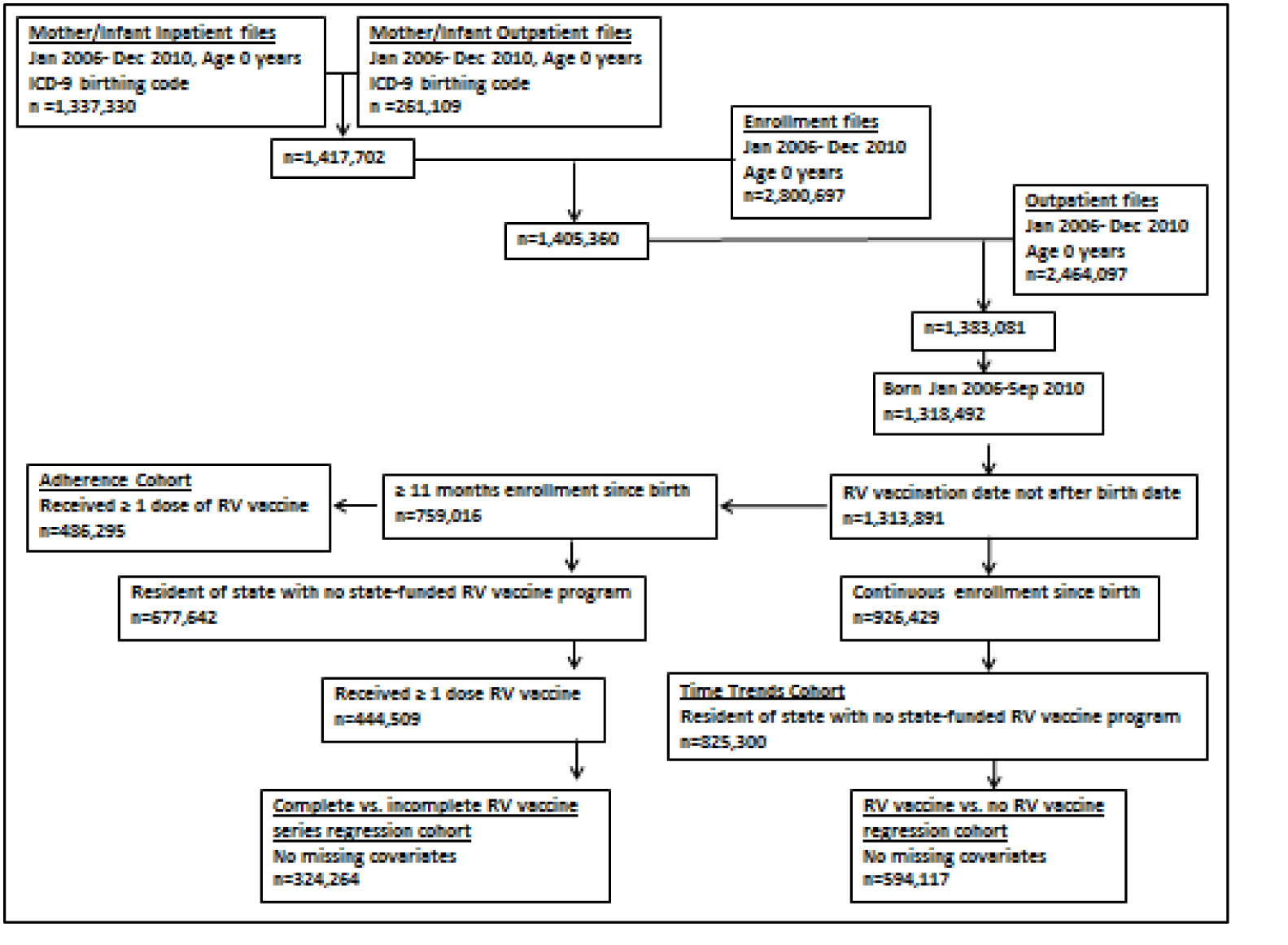

Supplement: Figure S1 — Development of study cohorts, MarketScan Research. Databases, 2006–2010 Abbreviations: Dec, December; ICD-9, International Classification of Diseases, Ninth Revision; Jan, January; RV, rotavirus; Sep, September. (TIF) [file pone.0073825.s001.tif]
